# A novel combined pacing and pattern matching approach for identifying critical isthmuses in reentrant atrial tachycardia

**DOI:** 10.1016/j.hrcr.2025.03.020

**Published:** 2025-03-28

**Authors:** Daisuke Munakata, Tomoyuki Uchida, Takeshi Ueyama, Yasuhiro Ikeda

**Affiliations:** 1 Department of Clinical Engineering, Yamaguchi Prefectural Grand Medical Center, Yamaguchi, Japan; 2 Department of Cardiology, Yamaguchi Prefectural Grand Medical Center, Yamaguchi, Japan

**Keywords:** Macroreentry, Mapping technique, Localized reentry, Ablation, iPASO

**Key Teaching Points**

• The Critical Isthmus Orientation With Mapping and Pacing Score method complements conventional techniques: By integrating Intracardiac Pattern-Match Score mapping with conventional pace mapping techniques, the accuracy of identifying complex atrial tachycardia circuits is improved. This new approach enhances circuit identification, contributing to the identification of isthmuses.
• Importance of numerical indicators in identifying local reentry circuits: The calculation of isthmus ratio provides a standardized measurement method for objectively evaluating potential isthmus locations. This index enables comparison between different sites and improves the accuracy of identifying true circuit components.
• Value of multifaceted evaluation in complex tachycardia circuits: Rather than relying on a single mapping method, combining Intracardiac Pattern-Match Scores, pace mapping, and entrainment analysis improves the localization of isthmuses, especially in anatomically complex regions. This may increase the efficiency and success rate of ablation strategies.

## Introduction

The accurate identification of critical isthmuses in reentrant atrial tachycardia circuits remains a significant challenge in cardiac electrophysiology, despite recent technological advances in mapping systems. Conventional approaches using local activation time and voltage mapping have demonstrated clinical utility; however, they frequently encounter limitations when addressing complex circuits, particularly in regions characterized by low voltage or fractionated signals.^1^

The recent development of Intracardiac Pattern-Match Scoring (iPASO) mapping represents a significant advancement in arrhythmia mapping, especially for identifying focal atrial tachycardia and premature atrial contraction origins.^2^ Although iPASO mapping has demonstrated remarkable efficacy in mapping focal arrhythmias, its application to reentrant circuits remains unexplored.

In this case report, we present Critical Isthmus Orientation With Mapping and Pacing Score (COMPAS), a novel methodology that integrates iPASO score matching with established pace mapping techniques for ventricular tachycardia.^3^^,^^4^ This innovative approach combines the pattern matching capabilities of iPASO technology with the spatial and temporal information derived from pace mapping. Through integration of these complementary mapping modalities using the CARTO 3 System (Biosense Webster, Diamond Bar, CA) equipped with the Intracardiac Pattern Matching module, we aimed to enhance the precision of critical isthmus identification within reentrant atrial tachycardia circuits, particularly in scenarios where traditional mapping approaches proved insufficient.

## Case report

A 79-year-old male patient was referred to our institution following the detection of atrial fibrillation during a routine medical examination. Initial evaluation revealed a regular tachycardia with a cycle length of 250 ms. After comprehensive informed consent was obtained, we performed an electrophysiological study under general anesthesia using the CARTO 3 V8 system (Biosense Webster) with an open-irrigated contact-force sensing catheter (ThermoCool SmartTouch ST-SF, Biosense Webster).

**Figure 1:**
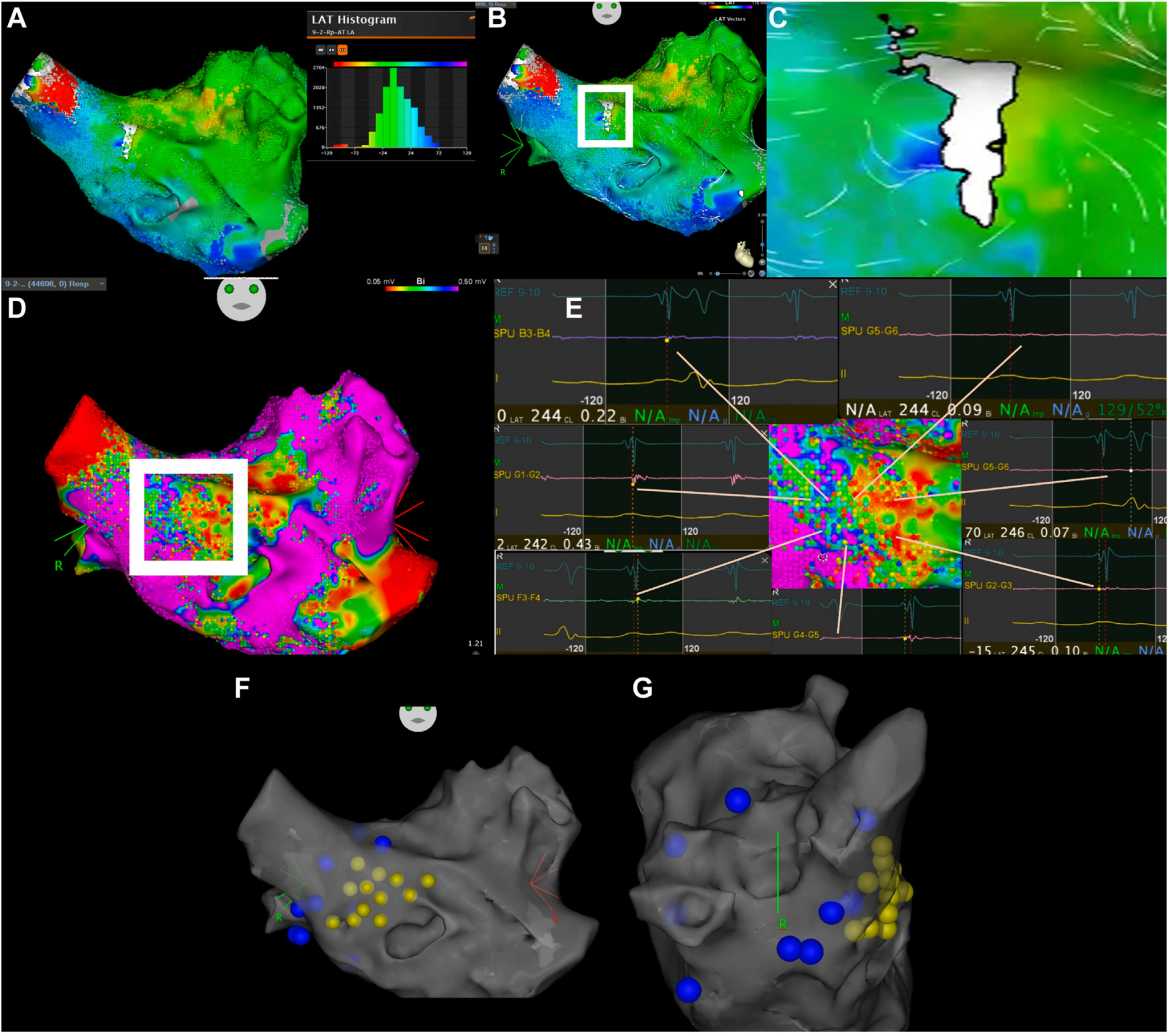
**A:** Conventional local activation time (LAT) map showing unclear activation pattern during tachycardia with corresponding LAT histogram revealing the continuous activation pattern characteristic of reentry. **B, C:** Local activation time velocity vector analysis defining a localized reentrant circuit with *arrows* indicating propagation direction around the circuit. **D:** Bipolar voltage map highlighting low-voltage substrate (*red areas*, <0.5 mV) corresponding to the anatomic substrate of the reentrant circuit. **E:** Fractionation map showing regions with complex fractionated atrial electrograms that correlate with the functional conduction pathway within the critical isthmus. **F:** Anteroposterior view of post-pacing interval (PPI) mapping. *Yellow tags* indicate PPI-tachycardia cycle length (TCL) ≤30 ms (within circuit); *blue tags* indicate PPI-TCL >30 ms (outside circuit). **G:** Right lateral view of PPI mapping demonstrating spatial distribution of measurements.

Surface electrocardiograms and bipolar intracardiac electrograms were continuously recorded using a BC-2100 system (Fukuda Denshi, Tokyo, Japan). To establish stable reference signals for mapping, we positioned a 6F 20-pole electrode catheter (BeeAT, Japan Lifeline, Tokyo, Japan) in the coronary sinus. High-density activation mapping revealed a localized reentry pattern in the area (Figure 1A–1E).

The electroanatomical mapping demonstrated characteristics suggestive of a localized reentrant circuit, a pattern that conventional mapping techniques often struggle to delineate accurately. Post-pacing interval measurements showed consistent entrainment with concealed fusion in the region anterior to the right superior pulmonary vein (RSPV) (Figure 1F and 1G). Coronary sinus ostium, coronary sinus distal, and high right atrium did not match.

Local activation time velocity vector analysis of the RSPV anterior region revealed a characteristic localized reentry pattern (Figure 1C and 1E), further supporting the presence of a localized reentrant mechanism. The conduction pattern demonstrated slow conduction zones consistent with areas of anatomic or functional block (Figure 1C).

### COMPAS mapping technique

The pacing cycle length was set at 10–20 ms shorter than the tachycardia cycle length, with output ranging from 2 to 10 V adjusted to achieve consistent capture, and a fixed pulse width of 0.5 ms.

**Figure 2:**
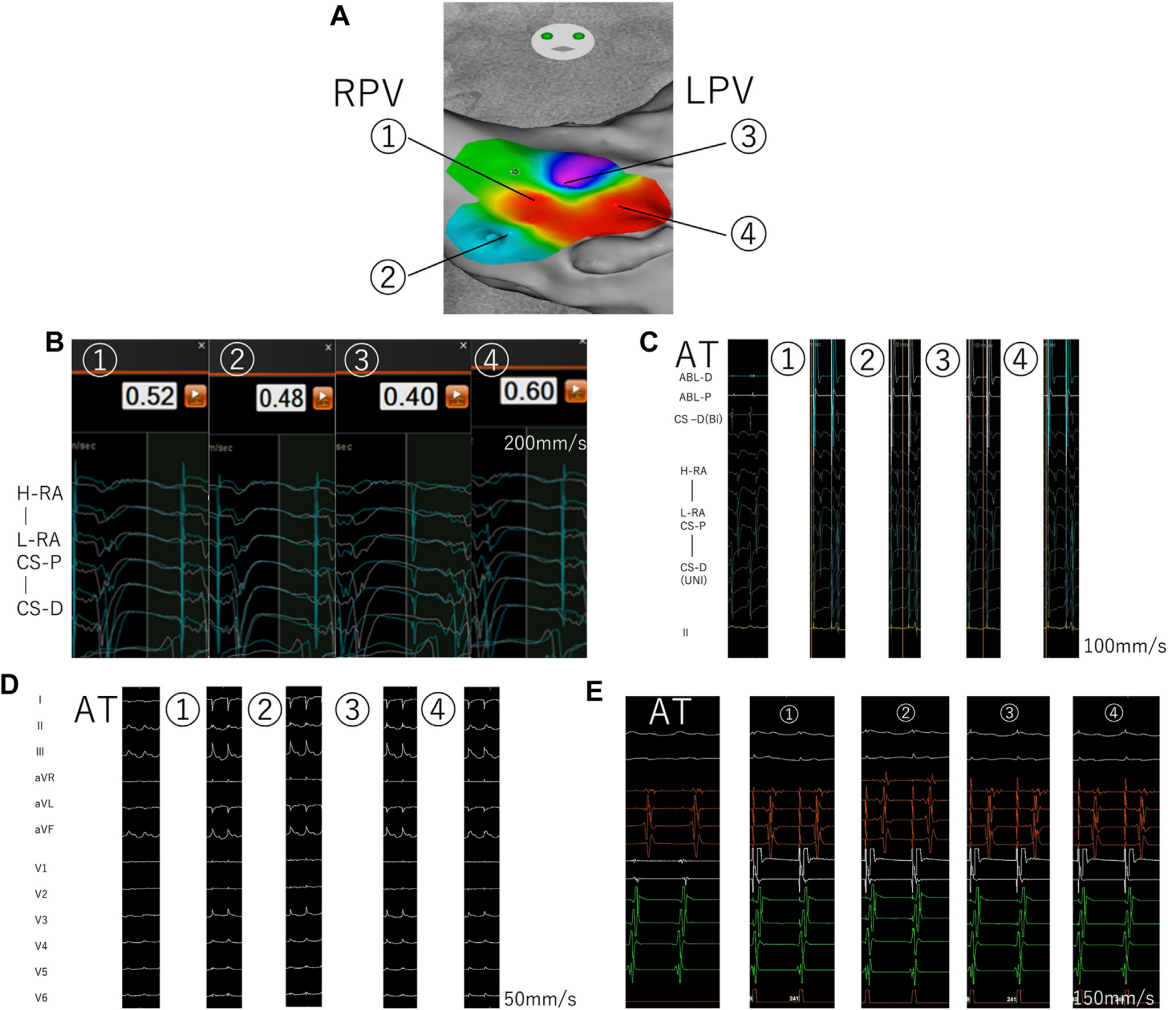
**A:** Spatial distribution of Intracardiac Pattern-Match Scoring (iPASO) scores showing color-coded correlation with the tachycardia circuit, demonstrating concordance with traditional entrainment mapping findings. **B:** Quantitative analysis of iPASO scores across all mapped sites, with threshold values indicating critical circuit components. **C:** CARTO TIMELINE analysis revealing temporal correlation patterns between paced sequences and tachycardia activation, confirming circuit participation. **D:** Comparative analysis of P-wave morphologies during tachycardia vs pacing maneuvers, showing morphologic concordance at sites within the circuit. **E:** Sequential bipolar pacing configuration analysis demonstrating site-specific local activation patterns that validate circuit participation and exit sites.

The mapping process integrated multiple complementary approaches to identify potential isthmus sites. Initially, we conducted systematic pacing throughout the chamber of interest, focusing on regions demonstrating post-pacing interval <30 ms from the tachycardia cycle length (Figure 1F and 1G). At these identified sites, we performed simultaneous iPASO mapping to assess morphologic correlation between paced and tachycardia activation patterns (Figure 2A and 2B). To validate our findings, we analyzed the activation sequence using the CARTO TIMELINE, which demonstrated high morphologic similarity between paced and tachycardia sequences (Figure 2C). In addition, we compared P-wave morphologies during pacing with those observed during tachycardia to confirm concordance of activation patterns (Figure 2D).^5^

**Figure 3:**
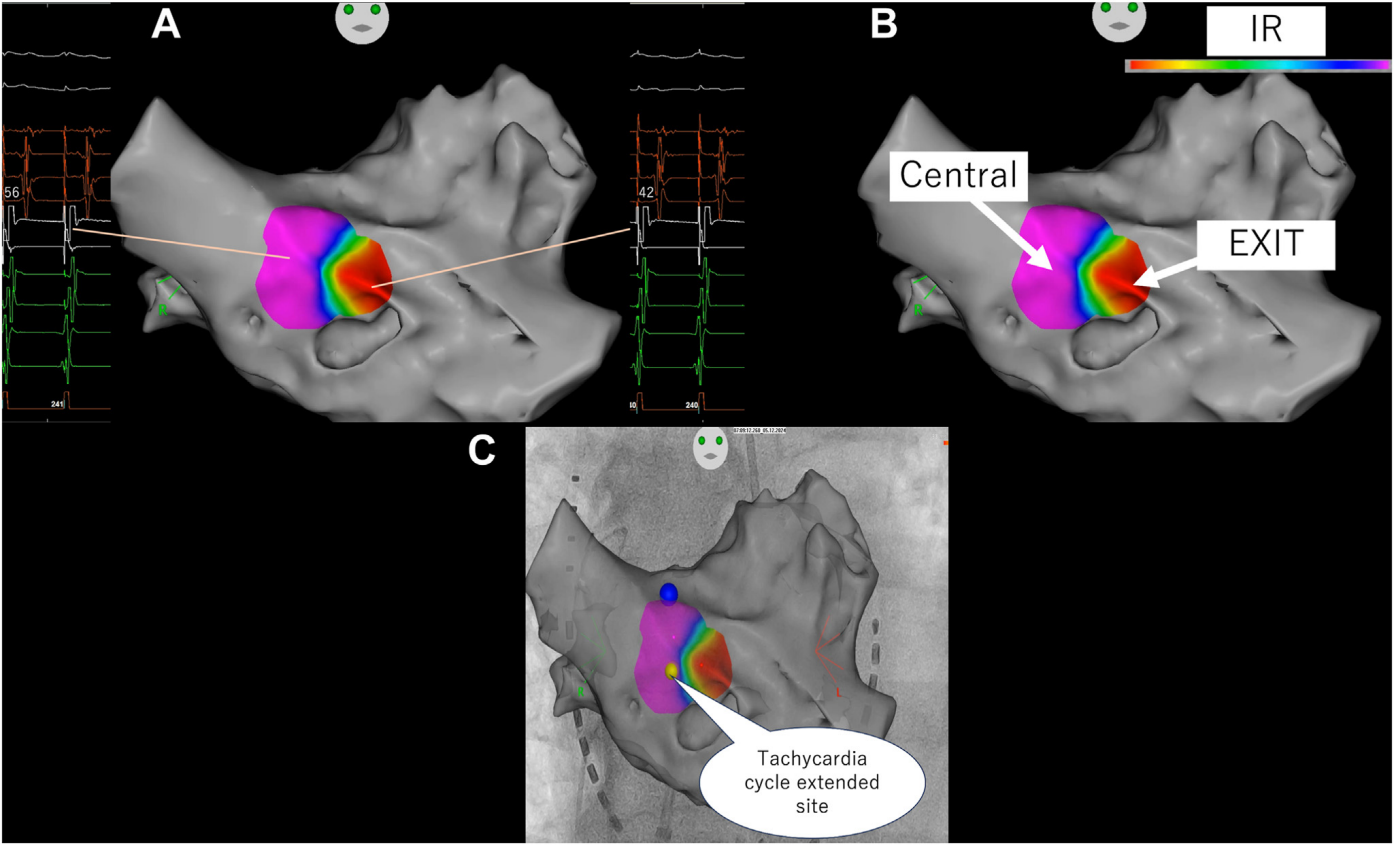
**A:** Bipolar voltage map overlaid with stimulus-to-P-wave intervals in regions with high Intracardiac Pattern-Match Scoring (iPASO) scores (>60), demonstrating the relationship between substrate characteristics and activation timing within the critical isthmus. **B:** Color-coded representation of isthmus ratio (IR) demonstrating the spatial distribution of IR values and identifying potential isthmus locations. **C:***Yellow tag* indicates sites where tachycardia extended and *blue tag* represents sites where tachycardia terminated during ablation.

For locations exhibiting iPASO scores >60%, we calculated the isthmus ratio, as shown in the equation. Recognizing the inherent challenges in precisely determining P-wave onset in complex atrial arrhythmias, we used the activation timing of high right atrial electrodes 1–2 as stable reference points (Figures 2E and 3A). The isthmus ratio was calculated using the following formula:

$$\begin{array}{rr} IR & =-\left(100-\frac{\text{STIM}-\text{HRA}\;3,4\;\text{wave}\;\text{onset}}{\text{tachycardia}\;\text{cycle}\;\text{length}}\times 100\right)\;\text{ms} \end{array}$$

This approach provides a standardized measure for comparing different potential isthmus sites. Manual annotation adjustments were performed within a temporal window of –100 ms to 0 ms relative to the reference signal, ensuring precise alignment of activation timing. This methodology allowed for consistent evaluation of potential isthmus sites, while accounting for local activation characteristics and timing relationships.

## Results

Implementation of the COMPAS mapping technique successfully identified a localized reentrant circuit within the left atrium. Using this novel approach, we precisely characterized the isthmus. The central site was identified at the ostium of the RSPV, with the exit site located in the anterior-middle region (Figure 3B).

Initially, pulmonary vein isolation was performed. Initial radiofrequency application targeted the identified central site (Figure 3C), resulting in significant electrophysiological changes: the tachycardia cycle length increased from 250 ms to 350 ms, accompanied by marked changes in activation sequence. These observations suggested transformation from a localized reentrant circuit to a broader macroreentrant circuit involving the entire left atrium.

Subsequent ablation targeting the RSPV roof resulted in immediate tachycardia termination. This response pattern indicated that the initial circuit likely represented a localized reentry mechanism maintaining the rapid 250-ms cycle length, which, following the first ablation, transformed to use an alternative pathway through the RSPV roof.

## Discussion

This case demonstrates the potential clinical utility of the COMPAS mapping technique in addressing complex arrhythmia substrate characterization. The integration of iPASO with conventional mapping techniques enhanced our ability to precisely identify critical isthmuses in anatomically challenging locations. Our experience suggests several advantages over traditional mapping approaches. The enhanced accuracy through multimodal mapping integration enables comprehensive arrhythmia substrate characterization, while improving our ability to delineate complex reentrant circuits in areas of low voltage or fractionated signals. This approach also offers potential for standardized mapping protocols across different operators and institutions, with real-time validation of circuit components through simultaneous assessment of pattern matching and pacing responses.

### Limitations

Several important limitations warrant consideration. As a single case report, these findings require validation through larger-scale studies across diverse patient populations. The technique involves careful interpretation of pacing artifacts, requiring expertise to ensure accurate analysis and avoid misinterpretation.

Technical considerations include the impact of reference stability on iPASO scoring. In this case, despite initially acceptable scores, we observed gradual deterioration, with a maximum unpaced iPASO score of 75 points, potentially reflecting minor displacement of the coronary sinus catheter reference position.

A notable limitation emerged in our inability to clearly delineate the entrance site, despite successful exit site identification. This observation suggests that atrial circuits may possess shorter isthmuses compared with ventricular circuits, particularly in cases of localized reentry. In addition, the circuit characteristics suggested involvement of both endocardial components and potential participation of the vein of Marshall in the macroreentrant pathway, indicating areas for future methodological refinement.

## Conclusion

The COMPAS mapping technique represents a promising approach for identifying critical isthmuses in localized reentrant AT, particularly in regions characterized by low voltage or fractionated electrograms. By integrating iPASO pattern matching with established pacing methodologies, this approach provides enhanced spatial and temporal resolution for mapping complex arrhythmia circuits. This case demonstrates the potential utility of COMPAS in delineating localized reentry mechanisms and guiding ablation strategy. The observed transformation from localized reentry to macroreentry after initial ablation highlights the complexity of atrial arrhythmia substrates and underscores the importance of comprehensive mapping approaches. Further prospective studies are warranted to validate these findings across diverse patient populations and arrhythmia mechanisms. Continued refinement of the methodology may enhance its applicability to various arrhythmia subtypes and anatomic locations. The standardization of this approach could potentially improve procedural outcomes and facilitate knowledge transfer in the electrophysiology community.

## Disclosures

The authors have no conflicts of interest to disclose.
